# Jasmonates in plant growth and development and elicitation of secondary metabolites: An updated overview

**DOI:** 10.3389/fpls.2022.942789

**Published:** 2022-08-15

**Authors:** Soo-In Sohn, Subramani Pandian, Kasinathan Rakkammal, Muthiah Joe Virgin Largia, Senthil Kumar Thamilarasan, Sekaran Balaji, Yedomon Ange Bovys Zoclanclounon, Jayabalan Shilpha, Manikandan Ramesh

**Affiliations:** ^1^Department of Agricultural Biotechnology, National Institute of Agricultural Sciences, Rural Development Administration, Jeonju, South Korea; ^2^Department of Biotechnology, Alagappa University, Karaikudi, Tamil Nadu, India; ^3^Department of Botany, St. Xavier’s College, Palayamkottai, Tamil Nadu, India; ^4^Independent Researcher, Madurai, Tamil Nadu, India; ^5^Department of Biotechnology, School of Life Sciences, Pondicherry University, Puducherry, India

**Keywords:** methyl jasmonate, jasmonic acid, plant growth, elicitation, secondary metabolites, medicinal plants

## Abstract

Secondary metabolites are incontestably key specialized molecules with proven health-promoting effects on human beings. Naturally synthesized secondary metabolites are considered an important source of pharmaceuticals, food additives, cosmetics, flavors, etc., Therefore, enhancing the biosynthesis of these relevant metabolites by maintaining natural authenticity is getting more attention. The application of exogenous jasmonates (JAs) is well recognized for its ability to trigger plant growth and development. JAs have a large spectrum of action that covers seed germination, hypocotyl growth regulation, root elongation, petal expansion, and apical hook growth. This hormone is considered as one of the key regulators of the plant’s growth and development when the plant is under biotic or abiotic stress. The JAs regulate signal transduction through cross-talking with other genes in plants and thereby deploy an appropriate metabolism in the normal or stressed conditions. It has also been found to be an effective chemical elicitor for the synthesis of naturally occurring secondary metabolites. This review discusses the significance of JAs in the growth and development of plants and the successful outcomes of jasmonate-driven elicitation of secondary metabolites including flavonoids, anthraquinones, anthocyanin, xanthonoid, and more from various plant species. However, as the enhancement of these metabolites is essentially measured *via in vitro* cell culture or foliar spray, the large-scale production is significantly limited. Recent advancements in the plant cell culture technology lay the possibilities for the large-scale manufacturing of plant-derived secondary metabolites. With the insights about the genetic background of the metabolite biosynthetic pathway, synthetic biology also appears to be a potential avenue for accelerating their production. This review, therefore, also discussed the potential manoeuvres that can be deployed to synthesis plant secondary metabolites at the large-scale using plant cell, tissue, and organ cultures.

## Introduction

Plants produce a variety of low molecular weight organic compounds, which are classified as primary or secondary metabolites. It requires primary metabolites for growth and development, whereas secondary metabolites serve as defence molecules that protect plants from adverse conditions (Verpoorte et al., 2000; Afrin et al., 2015). Secondary metabolites are produced by plants in their roots, stems, leaves, and other aerial regions with therapeutic beneficial, and have been utilized to treat many diseases since time immemorial (Pan et al., 2014; Raomai et al., 2015). The majority of secondary metabolites are obtained from wild plants, and that consequences their over-exploitation to eventual extinction. Chemical synthesis generates massive quantities of secondary metabolites, minimizing the need of their extraction from plants. However, the structure of many secondary metabolites is either unknown, extremely complicated, or their metabolic pathways are unknown. Consumers always prefer naturally derived products rather than chemically derived counterparts (Namdeo, 2007). However, the synthesis of secondary metabolites in a plant is relatively low and highly depending on the physiological and developmental stages of that plant (Thakur et al., 2013). To resolve this issue, a variety of *in vitro* techniques have widely been used to stimulate the production of bioactive secondary metabolites. Under natural conditions, secondary metabolites can be exogenously induced by plant hormone substances (Zhao et al., 2005; Ji et al., 2019). Accordingly, many biotechnological approaches have been introduced and explored for enhancing the production of secondary metabolites from medicinal plants. Among a few of the techniques used are cell line screening, elicitation, precursor feeding, hairy root culture, biotransformation, and others (Namdeo, 2007; Datta et al., 2011). Although many plant species have successfully been propagated in cell cultures, not all of them produce adequate secondary metabolites. When comparing with other techniques, treating undifferentiated cells by elicitors, such as methyl jasmonates (MJ), salicylic acid (SA), and chitosan might often accelerate the synthesis of secondary metabolites in most plants (Khanam et al., 2022).

**Figure 1:**
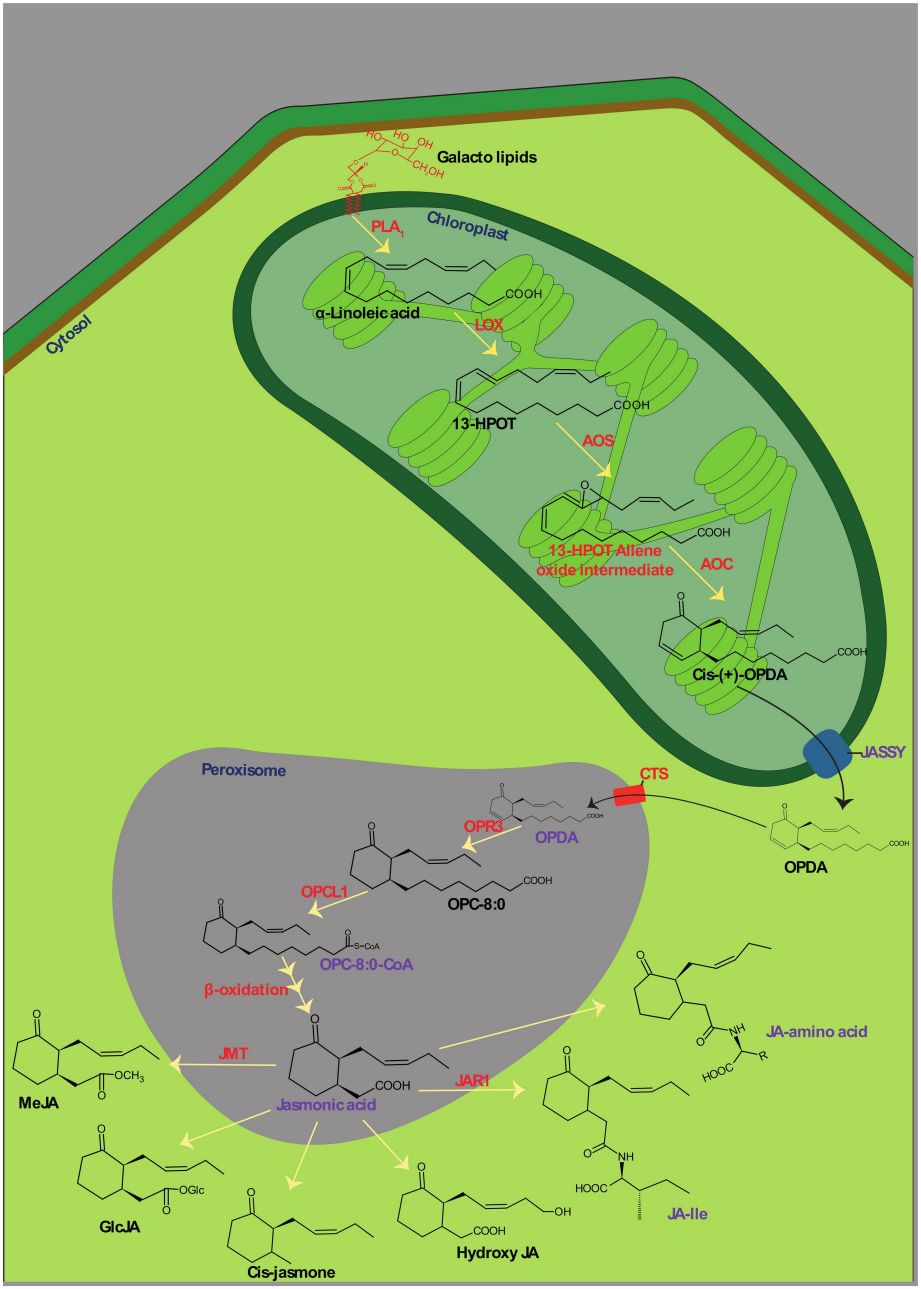
Biosynthesis of jasmonic acid (JA) and its direct derivatives. PLA_1_, Phospholipase A1; LOX, 13-lipoxygenase; HPOT, 13S-hydroperoxyoctadecatrienoic acid; AOS, Allene oxide synthase; AOC, Allene oxide cyclase; OPDA, Oxophytodienoic acid; OPR3, OPDA reductase; OPC-8:0, 3-oxo-2(29-[Z]-pentenyl)cyclopentane-1-octanoic acid; OPCL1, OPC-8:0 CoA ligase 1; JMT, Jasmonic acid carboxyl methyltransferase; JAR1, Jasmonate resistance-1; MeJA, Methyl jasmonate; GlcJA, Jasmonic acid Glycosyl ester; JA-Ile, Jasmonoyl-L-isoleucine.

Jasmonates is the collective term for jasmonic acid and its derivatives. It was originally isolated from the essential oil of *Jasminum grandiflorum* (Demole et al., 1962). JAs are cyclopentanone molecules generated from α-linolenic acid. It is the major precursor of several chemicals in this category, such as MJ (Ho et al., 2020). The biosynthesis of JAs has hitherto been explored in several monocotyledonous and dicotyledonous plants by having *Arabidopsis thaliana* and *Solanum lycopersicum* as model plants (Ruan et al., 2019). It was discovered with two phases of synthesis, *viz.*, the formation of an intermediate, oxophytodienoic acid (OPDA), and biosynthesis of JAs and their derivatives (Figure 1). Jasmonate synthesis commences when the Phospholipase A1 (PLA1) mediated release of α-linolenic acid (α-LeA) from the sn1-position of plastid membranal galactolipids and which endows the intermediate product, OPDA through sequential steps mediated by several enzymes of the plastid (Wasternack and Song, 2017). In an initial step, the released α-LeA is oxygenated to 13S-hydroperoxy-octadecatrienoic acid (13-HPOT) by an enzyme, 13-lipoxygenase (13-LOX). Hitherto, four known 13-LOXs (LOX2, LOX3, LOX4, and LOX6) have been elucidated with tissue-specific roles including the synthesis of jasmonate in plants and are thereby predominantly involved in wound healing (Caldelari et al., 2011; Wasternack and Hause, 2013). Subsequently, Allene oxide synthase (AOS) catalyzes the conversion of 13-HPOT into an intermediate, 13-HPOT-allene oxide and further cyclization reaction in the hydrocarbon chain of HPOT by Allene oxide cyclase (AOC) ends up in the final intermediate of phase-I, OPDA. Allene oxide intermediates can also be converted into α-and β-ketol *via* spontaneous hydrolysis (Lu et al., 2014). The second phase of jasmonate synthesis is carried out in peroxisomes. For jasmonate synthesis, plant cells need to traffic plastid-synthesized OPDA into peroxisomes. OPDA is required to be exported from plastids and imported into peroxisomes. Though the influx mechanisms of OPDA to peroxisomes are well understood, the efflux of the protein through double-membraned plastids has long been a mystery. In a recent finding, Guan et al. (2019) discovered a protein called JASSY with the activity of OPDA effluxion. JASSY, a plastid outer membranal protein, has been implicated in OPDA effluxion out of the plastid in several mechanistic studies (Simm et al., 2013). However, none of the studies described the specificity of OPDA towards JASSY (Wasternack and Hause, 2019). The exported OPDA can be influxed into peroxisomes from the cytosol by either ATP-dependent Binding Cassette protein, COMATOSE (CTS) or ion-trapping mechanisms (Theodoulou et al., 2005). The knockout mutant of either of these two transporters perturbs jasmonate synthesis but not OPDA formation (Guan et al., 2019), indicating their auspicious role in OPDA trafficking. In peroxisomes, OPDA is first reduced by OPDA reductase (OPR3) to 3-oxo-2-[Z]-(pentenyl)-cyclopentane-1-octanoic acid (OPC-8: 0). Subsequently, carboxyl-CoA is ligated by OPC-8: 0 CoA ligase 1 (OPCL1) at the carbonyl end of OPC-8:0, which produces OPC-8:0-CoA. OPC-8:0-CoA further undergoes β-oxidation thrice, which shortens its hydrocarbon chain at the carbonyl end and gives rise to the final product of Jasmonic acid (Sanders et al., 2000; Andersson et al., 2006; Lu et al., 2014). Several derivatives are then formed from jasmonic acid, among which MJ and jasmonoyl-L-isoleucine (JA-Ile) exhibit profound effects on plant physiology. MJ and JA-Ile are formed through the catalyzed reaction of enzymes, Jasmonic acid carboxyl methyltransferase (JMT) and Jasmonate resistance-1 (JAR1), respectively. These derivative reactions are deployed in the cytoplasm of plant cells (Ruan et al., 2019).

**Figure 2:**
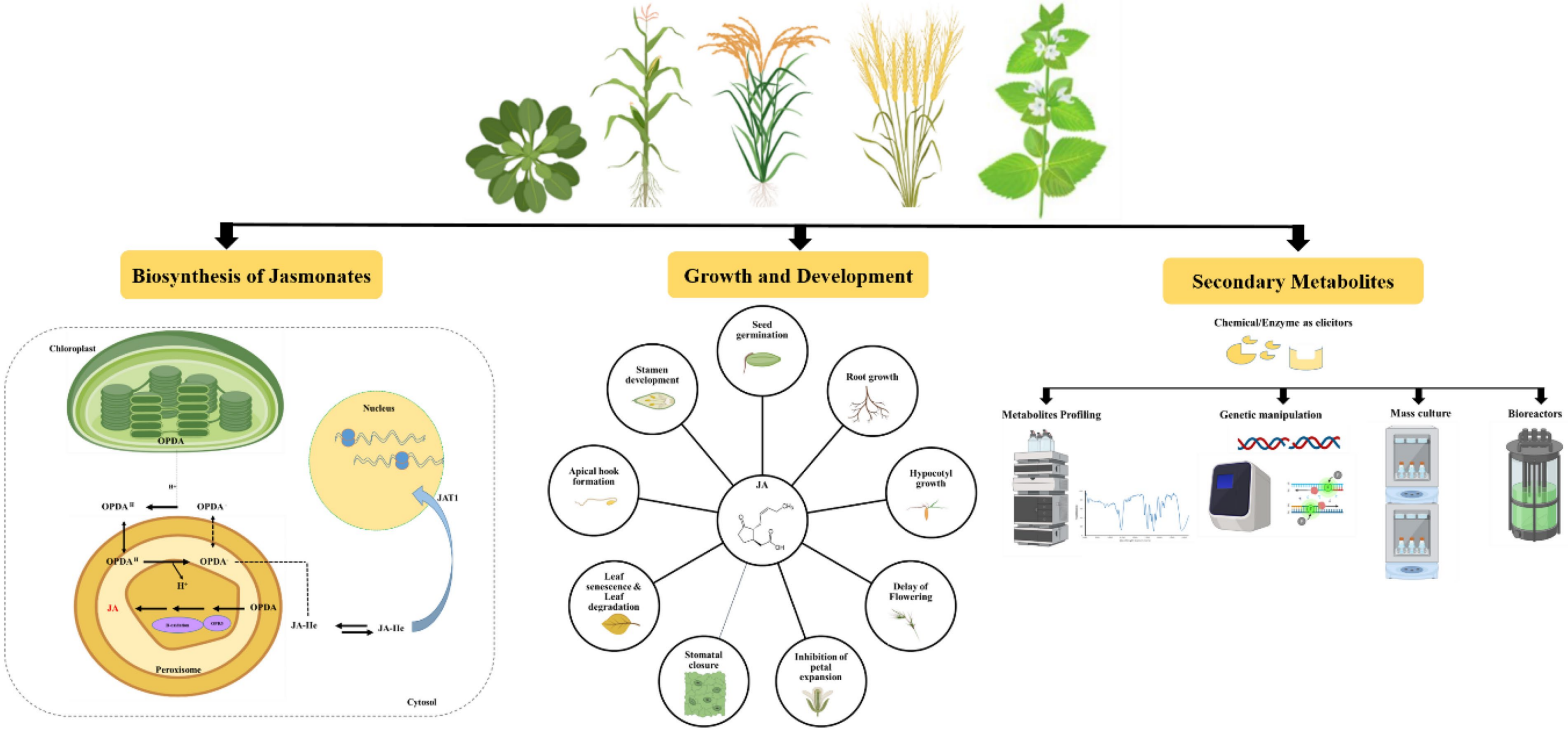
Schematic representation of jasmonates biosynthesis, role in growth and development and elicitation of secondary metabolites.

Jasmonates can coordinate a lot of cellular activities, including plant growth and development and regulation of plant responses to biotic and abiotic stresses (Afrin et al., 2015). They are also involved in floral development, fruit ripening, tendril coiling, potato tuberization, trichome formation, and arbuscular micorrhizal fungi association with plants (Browse, 2005; Balbi and Devoto, 2008; Reinbothe et al., 2009; Yoshida et al., 2009). From gymnosperms to angiosperms, they act as unique and conserved elicitors for the production of secondary metabolites (Zhao et al., 2005; Pauwels et al., 2009). The elicitation process induces the crosstalk between JAs and their receptors in the plasma membrane. It also triggers a cascade of defence responses in the cells, including the production of reactive oxygen and nitrogen species (ROS and RNS) and the induction of oxidative stress-protective enzymes (Giri and Zaheer, 2016). This results in the synthesis and accumulation of signaling molecules such as JA, SA, nitric oxide (NO), and ethylene (ET), as well as the regulation of secondary metabolite biosynthesis gene expression (Zhao et al., 2005; Baenas et al., 2014; Rahimi et al., 2015). Several excellent reviews on JAs role in plant development, immunity, and abiotic stress tolerance have recently been published (Yan and Xie, 2015; Sharma and Laxmi, 2016; Wasternack and Strnad, 2016; Huang et al., 2017; Nguyen et al., 2022). In this review, we provide updated information on the mechanisms of action of JAs in plant growth and development and further elaborate on their role in the elicitation of secondary metabolites in important medicinal plants (Figure 2).

## Regulation of plant growth and development

**Figure 3:**
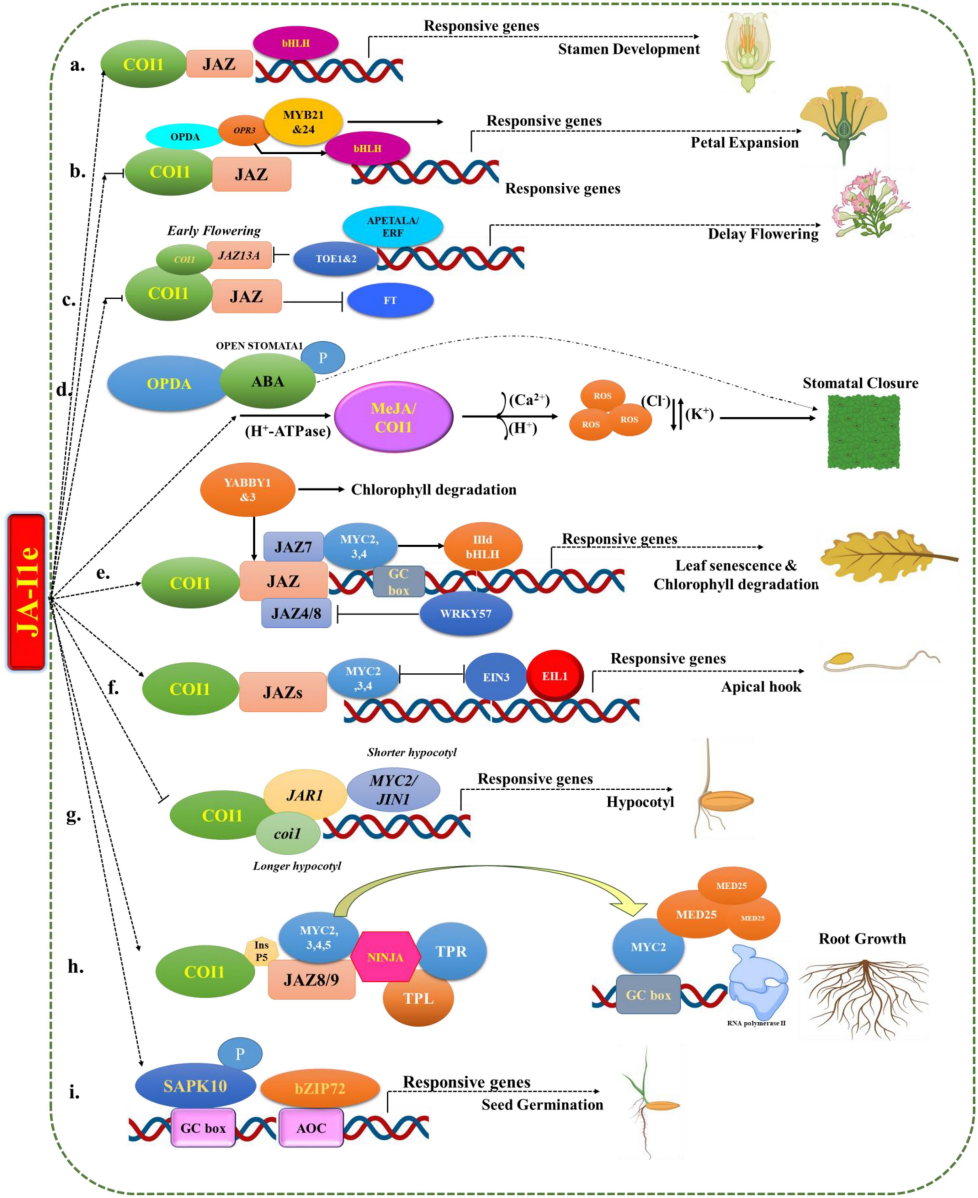
Schematic diagram for role of jasmonates in the plant growth and development.

Jasmonates are lipid-derived hormones that act synergistically with endogenous hormones in response to environmental signals to regulate plant growth, development and defence (Figure 3). In the last decade, research has been carried out to examine the role of JA in plant maturation and development (Ghorbel et al., 2021). According to previous studies, JAs are involved in a variety of plant expanding processes, including root primary growth, leaf senescence, and reproductive development (Wasternack, 2007; Kim et al., 2015).

### Effects on seed germination

Phytohormones such as ABA (Abscisic acid), IAA (Indoleacetic acid), and JA have been shown to promote seed germination (Xiao et al., 2018). Seed germination is suppressed by both ABA and JA, but their interactions during this process are unknown (Tang et al., 2020). In *Arabidopsis*, JA inhibition occurs without the involvement of the *COI1* co-receptor (Dave et al., 2011). Cold-stimulated seeds germination resulted in an increase in endogenous JA after overexpression of JA biosynthesis-related genes in *Triticum aestivum* plants, so JA promotes cold-stimulated germination (Xu et al., 2015b; Avramova, 2017). A novel SAPK10-bZIP72-AOC’ rice pathway has recently been found, in which ABA stimulates the production of JA, which synergistically suppresses rice seed germination (Wang et al., 2020). Auto-phosphorylation activates *SAPK10* (Serine/threonine-protein kinase), which stabilizes *bZIP72* transcription factor (TF; Basic leucine zipper) and improves its binding to the G-box cis-element of the *AOC* (Assimilable organic carbon) promoter, boosting *AOC* transcription in the presence of high JA concentrations. Interestingly, ABA sensitivity was reduced after inhibiting JA biosynthesis (Wang et al., 2020).

### Inhibition of root growth

Plants with mutations in the JA-Ile (Jasmonoyl-isoleucine) COI1 (Coronatine-insensitive 1) co-receptor are resistant to the inhibition of primary root maturation caused by JAs (Yan et al., 2009). In addition, inositol pentakisphosphate (*InsP5*) enhances *COI1–JAZ9* (Jasmonate Zim Domain) interactions, which reduces the influence of JAs on root elongation and maturation (Mosblech et al., 2011). Coronatine-O-methyloxime is a competitive JA antagonist that suppresses coronatine inhibitory effect on primitive root growth by blocking COI1–JAZs interactions (Monte et al., 2014). The ERF (Ethylene-responsive element-binding factor) associated amphiphilic repression (EAR) domain is absent from the majority of JAZ proteins (13 members) in *Arabidopsis.* To crush JA responses, they should interact with TPL (TOPLESS) and TPL-related proteins (TPRs) *via* the EAR of NINJA proteins. JAZ8 and JAZ13, two non-canonical JAZ proteins, do not require NINJA (novel interactor of JAZ) proteins to interact with TPLs/TPRs, instead of using their single EAR domain directly (Thireault et al., 2015; Chini et al., 2016). Overexpression of NINJA proteins or modified JAZ proteins (containing a deletion or mutation in the Jas domain) decreased the inhibitory effect of JA on primary root development, which was surprising. However, the NINJA/TPL or combination mutations in JAZ7, JAZ8, JAZ10, and JAZ13 increased this inhibitory effect (Thireault et al., 2015; Thatcher et al., 2016). In *Arabidopsis*, bHLH (basic helix–loop–helix) transcription factors (MYC2 and its homologs MYC3/4/5) interact with JAZ proteins (Qi et al., 2015). At the primary root apex, MYC2/3/4 gives way to JAs, which inhibit primary root growth (Gasperini et al., 2015). MYC2 also lowers the activity of root meristematic cells and suppresses primary root growth by inhibiting the expression of *PLETHORA* genes (*PLT1* and *PLT2*; Chen et al., 2011). MYC2 also interacts with the MED25 (MEDIATOR25) subunit to inhibit RNA polymerase II, reducing its inhibitory action (Chen et al., 2011). MYC3–MED25 interactions have been demonstrated to be disrupted by JAZ9 (Gimenez-Ibanez et al., 2014). Inhibition of MYC2 ubiquitination and phosphorylation by PLANT U-BOX PROTEIN10 decreases JA-mediated root maturation inhibition in the presence of mitogen-activated protein kinase 3/6 (Chico et al., 2014; Sethi et al., 2014).

### Inhibition of hypocotyl growth

Jasmonate suppresses hypocotyl elongation in *Arabidopsis* under a variety of light stress conditions, including far-red and blue wavelengths *via* COI1 (Chen et al., 2013). JA-deficient mutant JAR1 (Jasmonic acid-resistant 1) plants cultivated under far-red light were found to have extended hypocotyls (Robson et al., 2010). Furthermore, either grown in the dark or under fared lights (Chen et al., 2013) or red light or a low R/FR light ratio, *coi1* mutants exhibit longer hypocotyls than WT plants (Robson et al., 2010; Chen et al., 2013). The MYC2/JIN1 (Jasmonate-insensitive mutant 1) mutant has a shorter hypocotyl when grown under far-red light and a low R/FR light ratio (Robson et al., 2010), but it has a longer hypocotyl when grown under blue light (Yadav et al., 2005). In rice, JA inhibits the maturity of coleoptiles and the growth of the plant. JA prevents the maturity of ear shoots in *Zea mays* (Yan et al., 2012; Yang et al., 2012).

### Delay of flowering

Jasmonate inhibits the transition from vegetative to reproductive maturity in *Arabidopsis*. Flowering is inhibited by the interaction of COI1 and JAZ. Indeed, both *coi1* mutant and JAZ13A transgenic plants showed early flowering. Furthermore, plant flowering is controlled by TARGET OF EAT TFs (TOE1 and TOE2), which are APETALA2/ETHYLENE RESPONSE FACTOR (AP2/ERF) domain TFs. In fact, their association with JAZ proteins limits blooming by inactivating *FLOWERING LOCUS T* transcription. Overexpression of TOE1 and/or TOE2 on the other hand, inhibits the *coi1* early-blooming phenotype (Zhai et al., 2015).

### Inhibition of petal expansion

Jasmonate suppresses petal growth in *Arabidopsis via* the COI1 pathway. In fact, when compared to wild type, *coi1* mutants have bigger petals at anthesis. The *aos* and *opr3* genes of *Arabidopsis* JA-deficient mutants showed a similar pattern (Brioudes et al., 2009; Reeves et al., 2012). MYB21 (Myeloblastosis 21) and MYB24 proteins are repressed by JA during the formation of sexual organs, resulting in petal growth restriction, indicating that MYB21 and MYB24 are crucial for petal expansion (Reeves et al., 2012). *MYB21* expression was shown to be higher in the petals of *aos* and *coi1* plants, resulting in continuous petal expansion and hence grand petals (Reeves et al., 2012). Furthermore, *opr3* plants have a higher level of bHLH TF BIGPETALp expression. This transcription factor regulates post-mitotic cell growth, resulting in expanded petals and cells (Brioudes et al., 2009).

### Regulation of stomatal closure

In plants, stomata aid in the regulation of gas exchange, water loss and the control of plant resistance to phytopathogens. JA is also involved in stomatal closure. In reality, Methyl-JA/COI1 activates an H^+^-ATPase in the plasma membrane. The depolarization of the membrane causes an influx of Ca^2+^ and an efflux of H^+^ (Yin et al., 2016). Methyl-JA/COI1 also causes the generation of reactive oxygen species (ROS), the stimulation of Cl^−^ channels (resulting in Cl^−^ efflux), and the stimulation of K^+^ efflux (through K^+^ channels), resulting in the loss of turgor guard cells and stomatal closure (Yan et al., 2015). Plants close their stomata in response to drought and salt stress. The activation of OPEN STOMATA1 protein kinase by JA and ABA pathways has been shown to modulate stomatal closure in *Arabidopsis* (Yin et al., 2016). Plants also generate OPDA rather than jasmonate in drought situations. In these circumstances, OPDA is more effective than JA. In *Arabidopsis*, OPDA interacts with ABA to improve stomatal closure (Savchenko et al., 2014).

### Induction of leaf senescence and chlorophyll degradation

In *Arabidopsis*, JA promotes leaf senescence. COI1 is required for this impact to occur (Qi et al., 2015). JAZ7 also found to inhibit leaf senescence in dark-grown plants (Yu et al., 2016). Many members of the NAC TF family (such as NAC019, NAC055, and NAC072) promote chlorophyll degradation by acting downstream of MYC2/3/4 (Melotto et al., 2006). Subgroup IIId bHLH TFs inhibit leaf senescence by binding competitively to their target promoters and inhibiting MYC2/3/4 activity (Qi et al., 2015). In the presence of JA, WRKY57 interacts physically with JAZ4/8 and acts as a negative regulator of leaf senescence (Jiang et al., 2014). The proteins YABBY1 and YABBY3 interact with JAZs, promoting chlorophyll degradation (Boter et al., 2015).

### Inhibition of apical hook formation

The COI1-JAZs-MYC2/3/4 cascade suppresses the development of an apical hook in dark-grown plants (Song et al., 2014). In dark stress circumstances, JA activates the transcription factors of MYC2, MYC3, and MYC4, which physically interact with and decrease the transcriptional activity of EIN3/EIL1 (Ethylene-insensitive3/ETHYLENE-INSENSITIVE3-like 1). Apical hook curvature is prevented and the *HOOKLESS1* gene, which controls apical hook formation, is downregulated (Song et al., 2014; Zhang et al., 2014). MYC2 also promotes the formation of EIN3 BINDING F-BOX PROTEIN1, which causes EIN3 degradation (Zhang et al., 2014).

### Stamen development in *Arabidopsis*

Many male sterile *Arabidopsis* plants have been reported, among which JA-deficient mutants such as *coi1, lox3* (Lipoxygenases)*, lox4, aos, opr3, fad3* (Acyl-lipid omega-3 desaturase)*, fad7, fad8, dad1* (Defective in anther dehiscence1), JAZ13A and JAZ10.4 mutations, as well as CYP94B3 (Jasmonoyl-isoleucine-12-hydroxylase) overexpression lines were discovered, all of which indicated impaired stamen development (Song et al., 2013). Exogenous administration of JA rescues stamen growth in plants deficient in JA production but not in JA signalling mutants, which is surprising (Jewell and Browse, 2016). Furthermore, in a *coi1* background, re-expressing COI1 in a variety of tissues, such as the filament epidermis or anthers, can restore anther dehiscence, filament elongation, and pollen maturation (Jewell and Browse, 2016). The R2R3-MYB TFs MYB21, MYB24, and MYB57 interact directly with JAZs (Song et al., 2011). Delayed anther dehiscence, a non-viable pollen grain, and short filaments are all symptoms of the *myb21 myb24* double mutant. Overexpression of MYB21 proteins in *coi1-1* plants results in restored stamen formation (Song et al., 2011). MYB21 and MYB24 regulate the stamen formation by physically connecting to MYC2, MYC3, MYC4, and MYC5 (Qi et al., 2015). Overexpression of MYC5 and MYC3 enhances stamen maturation and productivity in plants (Qi et al., 2015).

## Elicitation of secondary metabolites using jasmonates

Apart from their role in plant growth and development, JAs act as a major elicitor for the enhancement of secondary metabolites. Among the JAs, MJ has been extensively used as an elicitor to enhance a wide range of secondary metabolites for more than two decades. It has been reported to influence the production of phytochemicals in different *in vitro* culture systems, such as adventitious root culture, callus culture, multiple shoot culture, cell suspension culture, and hairy root culture (Nabi et al., 2021). MJ is the most frequently used elicitor, and it has been found to have a significant influence on secondary metabolite accumulation in plant cells and organs (Baenas et al., 2014; Giri and Zaheer, 2016). Exogenous application of MJ increases the concentration of phenols (Ahn et al., 2014), alkaloids (Zhou et al., 2015), terpenoids (Onrubia et al., 2013), coumarin (Dučaiová et al., 2016), anthocyanin (Ram et al., 2013) and polyamines (Cao et al., 2014), not only in plant cell cultures but also in whole plants (Ahn et al., 2014; Dučaiová et al., 2016; Ho et al., 2020). MJ was used as an effective elicitor in the root suspension of *Ajuga bracteosa*, leading to an increased phenolic and flavonoid content (Saeed et al., 2017). In addition, increased expression of genes and transcription factors related to secondary metabolite biosynthesis has also been reported. In recent years, elicitation of metabolites has been demonstrated in bioreactors as a prelude to large-scale commercial production of phytochemicals.

### Enhanced accumulation of high valued metabolites

#### In cell suspension cultures

A dramatic increase in rosmarinic acid content in cultured cells of *Lithospermum erythrorhizon* was observed after their exposure to MJ (Mizukami et al., 1993). MJ elicitation resulted in the induction of xanthones in cell suspension cultures of *Centaurium* spp. (Beerhues and Berger, 1995). A marked increase in the alkannin pigment content in cells and medium of suspension cultures of *Alkanna tinctoria* after treatment with MJ was observed (Urbanek et al., 1996). The addition of 0.5 μM MJ provoked a twofold to threefold increase in anthocyanin production over that of the control in cell cultures of *Vaccinium pahalae* (Fang et al., 1999). Supplementation of 100 μM MJ and 25 g/L sucrose produced 24 mg/L cephalomaninein in the cell suspension cultures of *Taxus chinensis* (Lan et al., 2002). Synthetic JAs, such as pentafluoropropyl jasmonate, 2-hydroxyethyl jasmonate and 2-hydroxyethoxyethyl jasmonate were found to promote ginsenoside production in cell suspension cultures of *Panax notoginseng* (Wang et al., 2005). A sixfold increase of phenolic compounds, flavanols and flavonols after JA elicitation was observed in *Hypericum perforatum* cell suspension cultures (Gadzovska et al., 2007). *Taxus cuspidata* var. nana cell suspension culture was reported to have a fourfold increased accumulation of paclitaxel, an anticancer diterpenoid, upon elicitation with 21 mg/L JA (Tachinbana et al., 2007). A concentration of 100 mg/L of MJ induced peruvoside production in *Thevetia peruviana* cell suspension cultures (Zabala et al., 2010). Suspension cultures of Habanero pepper exposed to MJ have led to the accumulation of capsaicinoids and vanillin (Gutiérrez-Carbajal et al., 2010). Sequential application of MJ, SA, and yeast extract to *Argemone mexicana* cell cultures resulted in a ninefold increase in sanguinarine accumulation over unexposed control cultures (Trujillo-Villanueva et al., 2012).

Leaf-derived cell culture of *Adhatoda vasica* elicited with 20 μM MJ resulted in a 3.7-fold higher yield of vasicine in comparison with control cultures (Bhambhani et al., 2012). The combined treatment with cyclodextrins (50 mM) and MJ (100 μM) resulted in enhancement of ajmalicine and catharanthine productivity and increased gene transcript accumulation in *Catharanthus roseus* cell cultures (Almagro et al., 2014). The combined treatment of UV-C and MJ highly induced the total intracellular stilbene production to its maximum in cell suspension cultures of *Vitis vinifera* L. (Xu et al., 2015a). MJ yielded the maximum gymnemic acid content of 135.41 ± 0.43 mg g^−1^ dry cell weight, after 72 h elicitor application in *Gynema sylvestre* cell suspension cultures (Chodisetti et al., 2015). Treatment of *Hypericum perforatum* cell cultures with 100 μM/L MJ on day 15, resulted in 2.7 times more flavonoid production (Wang et al., 2015). Ali et al. (2015) observed enhanced accumulation of total phenolic content, total flavonoid content, and the highest radical scavenging activity in suspension cultures of *Artemisia absinthium* treated with 1.0 mg/L of MJ, JA, and GA, each.

A synergistic combination of MJ (0.1 mM) and 2-hydroxypropyl-β-cyclodextrin (20 mM) increased the production of intracellular anthraquinones in suspension cultures of *Rubia tinctorum* (Perassolo et al., 2016). The ginsenoside biosynthesis-related genes and ginsenoside accumulation were highly induced by 100 μM MJ in combination with 200 μM of sodium nitro prusside in adventitious root cultures of *Panax ginseng* (Rahimi et al., 2016). A combination of 0.1 mM MJ and 0.1 mM SA in the immobilized cells of *Ginkgo biloba* increased the production of bilobalide and ginkgolides A, B, and C than in the unelicited cultures (Sukito and Tachibana, 2016). *In vitro* cell suspension culture of *Momordica dioica* elicited with JA produced higher amounts of flavonols, hydroxycinnamic acids, and hydroxybenzoic acids (Chung et al., 2017). The addition of 150 μM MJ enriched the yield of essential oil in *Coriandrum sativum* embryogenic cultures (Ali et al., 2019).

#### In callus cultures

The addition of 100 μM MJ increased the paclitaxel (an anticancer alkaloid) content from 2.37 to 90 μg g^−1^ and cephalomannine content from 5.14 to 29.14 μg g^−1^ (dry weight) in callus cultures of *Taxus* × media var. *Hatfieldii* (Furmanowa et al., 1997). Elicitation of the calli by MJ induced a 38% increase in total polyphenol production in *Eritrichium sericeum* (Inyushkina et al., 2007). Ram et al. (2013) found that 5 μM MJ promoted anthocyanin production in rose callus cultures. The contents of six naphthoquinone compounds were increased in the MJ-treated callus tissues of *Arnebia euchroma* and in particular, the bioactive component acetylshikonin nearly doubled its content due to MJ elicitation (Hao et al., 2014). In callus cultures of *Phyllanthus pulcher,* 1 mM of MJ resulted in the highest yield for total flavonoid and phenolic contents and antioxidant activity (Danaee et al., 2015). A significant increase in antioxidant activity was observed in the calli of three *Opuntia* species in media with 50 μM JA (Camarena-Rangel et al., 2017). Callus cultures of *Gardenia jasminoides* elicited by 200 μM MJ showed the maximum content of total chlorogenic acid (a polyphenolic antioxidant) and its derivatives and displayed a much higher antioxidant capacity (Liu et al., 2018).

#### In adventitious root cultures

Indole-3-butyric acid with MJ at 100 μM synergistically stimulated both root growth and ginsenoside accumulation in *Panax ginseng* adventitious root cultures (Kim et al., 2007). The growth of adventitious roots, the contents of triptolide and alkaloids were increased 1.04, 1.64 and 2.12-folds, respectively, when MJ was at 50 μM in adventitious root cultures of *Tripterygium wilfordii* (Li et al., 2015). Andrographolide (an antiviral diterpenoid) content of 10.8-fold was obtained after the first week of elicitation with 25 μM JA in adventitious root cultures of *Andrographis paniculata* (Zaheer and Giri, 2017). The maximum accumulation of flavonoids was induced on the third day with the addition of H_2_O_2_ combined with MJ in root cultures of *Stevia rebaudiana* (Alvarado-Orea et al., 2020).

#### In whole plant cultures

Treatment of *Glycyrrhiza glabra in vitro* plantlets with 0.1–2 mM MJ enhanced the production of glycyrrhizin, a saponin, by 3.8 times (Shabani et al., 2009). After 4 weeks of treatment with 0.025 mg/L of TDZ coupled with 0.1 mM MJ, the production of anti-inflammatory triterpenoids (madecassoside and asiaticoside) from whole plant cultures of *Centella asiatica* was found to be increased by 2.40-and 2.44-folds, respectively (Yoo et al., 2011). Shoot cultures of *Bacopa monnieri* elicited with a combination of 25 μM MJ and SA resulted in a fivefold increased accumulation of Bacoside A, a memory-boosting triterpenoid saponin (Largia et al., 2015). An increased accumulation of anti-inflammatory alkaloids (pteropodine, isopteropodine, speciophylline, rumberine, hameline and palmirine) was reported in JA elicited *Hamelia patens* (Flores-Sanchez et al., 2016). The highest dioscorealide B (a phenolic compound) contentwas recorded in the 100 μM JA elicited *in vitro* shoots of *Dioscorea membranacea* (Jirakiattikul et al., 2020).

#### In hairy root cultures

Biondi et al. (2000) demonstrated an enhancement in the levels of methyl putrescine, a polyamine in normal and hairy root cultures of *Hyoscyamus muticus* by using JA and MJ. MJ up-regulated the biosynthesis of sesquiterpene lactones in hairy root cultures of *Cichorium intybus* after 72 h of exposure (Malarz et al., 2007). In hairy root culture of *Taxus* × *media* var. *Hicksii,* the supplementation of 100 μM of phenylalanine together with 100 μM of MJ resulted in the enhancement of paclitaxel production from 40.3 to 568.2 μg L^−1^ (Syklowska-Baranek et al., 2009). The highest rhinacanthin (an antiviral naphthoquinone) content was observed after treatment with 10 μM MJ which was about 1.7 fold higher than control hairy root cultures of *Rhinacanthus nasutus* (Cheruvathur and Thomas, 2014). The elicitation of hairy roots of *Solanum trilobatum* with 4 μM MJ for 2 weeks boosted the accumulation of the alkaloid solasodine by 6.5-fold more than untransformed roots. They also noticed a significant improvement in total phenolics, total flavonoids and radical scavenging activity of MJ elicited hairy roots (Shilpha et al., 2015). *Isatis tinctoria* hairy root cultures elicited with 179.54 μM MJ caused 11-fold increased flavonoid production (Gai et al., 2019). The highest quantity of triterpenoids (60.25 mg/g DW) was produced in hairy root cultures of *Centella asiatica* treated with 400 μM MJ (Baek et al., 2020). A sneak peek of recent reports pertaining to jasmonate elicitation has been given in Table 1.

**Table 1:** Recent reports on the use of jasmonates for enhanced production of a variety of secondary metabolites through different *in vitro* culture systems.

Plant species	Type of culture	Jasmonates used	Increased effect on metabolites	Category of secondary metabolite	Reference
*Vitis vinifera*	cs	MJ + UV-C	64-fold higher resveratrol and 1,343-fold higher viniferin contents	Polyphenols	Wang et al. (2022)
*Tinospora cordifolia*	cs	100 μM MJ	5.57-fold higher than berberine content	Alkaloids	Pillai and Siril (2022)
*Digitalis purpurea*	cs	50 μM MJ + 100 mg/L spermidine	Increased TPC, antioxidant activities, cardenolides, and digitoxin contents	Steroids	Rad et al. (2022)
*Vernonia anthelmintica*	cs	0.8 mM MJ	2.2-fold higher accumulation of rhamnetin	Flavonoids	Rajan et al. (2020)
*Hyoscyamus muticus*	cs	100 μM MJ	Four times increased production of atropine	Alkaloids	Abdelazeez et al. (2022)
*Thevetia peruviana*	cs	3 μM MJ + 300 μM SA	Threefold increased dihydroquercetin and chlorogenic acid contents	Flavanoids	Mendoza et al. (2020)
*Oryza sativa*	cs	5 μM MJ	Enhanced production of resveratrol and piceid	Polyphenols	Kantayos et al. (2021)
*Salvia bulleyana*	hr	100 μM MJ	100% improvement in rosmarinic acid production	Polyphenols	Krzemińska et al. (2022)
*Cajanus cajan*	hr	MJ + cyclodextrin	277 fold higher Cajaninstilbene acid production	Stilbenes	Gajurel et al. (2022)
*Taxus* × *media*	hr	MJ	38% increased triterpenoids and steroids	Triterpenoids	Sykłowska-Baranek et al. (2022)
*Prunella vulgaris*	hr	100 μM MJ	2 times higher TPC, 2.4 times higher TFC and 1.7 times higher accumulation of rosmarinic acid	Polyphenols	Ru et al. (2022)
*Eclipta prostrata*	hr	100 μM JA	5.2-fold increase in wedelolactone, 1.6-fold increase in demethylwedelolactone and a 2.47-fold increase in 3,5-di-O-caffeoylquinic acid	Coumestans	Maciel et al. (2021)
*Allium jesdianum*	callus	MJ	Higher accumulation of TPC, TFC, total flavanols and anthocyanins	Anthocyanins	Yazdanian et al. (2021)
*Scutellaria laterifolia*	Whole plant	50 μM MJ	Improved biosynthesis of verbascoside	Flavonoids	Kwiecień et al. (2022)
*Astragalus membranaceus*	ar	200 μM MJ	2-fold higher calycosin-7-*O*-β-D-glucoside	Isoflavanoids	Feng et al. (2021)
*Salacia chinensis*	callus	75 μM JA	Increased production of TPC, TFC and mangiferin	Polyphenols	Chavan et al. (2021)
*Arachis hypogea*	hr	125 μM JA	Increased antioxidant potential of stilbenoid extracts		Gajurel et al. (2021)
*Senna obtusifolia*	hr	100 μM MJ	Enhanced production of betulinic acid	Triterpenoids	Kowalczyk et al. (2021)
*Citrullus colocynthis*	shoot	75 μM of MJ	Highest accumulation of cucurbitacin E	Triterpenes	Dasari et al. (2020)

MJ, Methyl Jasmonate; JA, jasmonic acid; cs, Cell suspension; hr, Hairy root; μM, Micro molar; SA, Salicylic acid; TPC, Total phenolic content; TFC, Total flavanoid content; ar, Adventitious root.

#### *In vivo* plants

Interestingly, a 40–70 fold increase in the level of furanocoumarins was observed in the leaves of *Apium graveolens* by the exposure of MJ vapours (Miksch and Boland, 1996). JA at 50 μM concentration for 4 days resulted in increased camptothecin (an alkaloid) production up to 11 times (Song and Byun, 1998). In two cultivars of *Ocimum basilicum*, foliar application of 0.5 mM MJ raised the percentages of linalool and 1,8-cineole (terpene alcohols) and increased their antioxidant activity (Talebi et al., 2018). The elicitation of Maritime and Monterey pine seedlings with 5 mM MJ resulted in increased total mono and sesquiterpenes, which led to increased resistance against pine weevil, *Hylobius abietis* (Lundborg et al., 2019). Foliar spray of JA (400 ppm) improved the accumulation of antidiabetic potential triterpenoids, withanolide A and withanolide B in *Withania somnifera* (Singh et al., 2020). *Punica granatum elicited with 200* μM MJ *displayed an increased accumulation of flavonols and phenols* (Chang et al., 2021). Bean varieties such as *Phaseolus vulgaris*, *Glycine max*, and *Vigna radiata* demonstrated a higher accumulation of isoflavanoids on MJ treatment (Gómez et al., 2022).

### Influence of jasmonates on gene transcripts

Elicitation treatment in general stimulates the production of secondary metabolites through the involvement of signal compounds. In the last decade, elicitation studies were blended with gene expression analysis through RT-PCR and qRT-PCR techniques to confirm the mechanism and mode of action of elicitation. MJ treatment resulted in a 50-fold induction of transcripts encoding the key triterpene biosynthetic enzyme β-amyrin synthase in *Medicago truncatula* cell suspension (Suzuki et al., 2005). Hairy root cultures of *Panax ginseng* elicited with MJ revealed the increased transcription of relevant responsive genes such as squalene synthase, squalene epoxidase, and dammarenediol synthase-II (Kim et al., 2009). In case of 65-day-old plantlets of licorice treated with MJ (0.1, 1 and 2 mM) exhibited increased expression of two key biosynthetic enzymes for terpenoid biosynthesis such as squalene synthase and beta-amyrin synthase (Shabani et al., 2010).

Methyl jasmonates application resulted in induction of pathogenesis-related genes in two cultivars of *Gossypium hirsutum* (Zambounis et al., 2012). MJ treatment increased the transcript levels of terpene biosynthesis genes such as 3-hydroxy-3-methylglutaryl-coenzyme A reductase, 1-deoxy-D-xylulose-5-phosphate reductoisomerase, and hydroxy-2-methyl-2-(E)-butenyl 4-diphosphate reductase, as well as parthenolide biosynthetic genes such as germacrene A synthase, germacren in *Tanacetum parthenium* (Majdi et al., 2015). Treatment of *Solanum trilobatum* hairy roots with 4 μM MJ has upregulated the expression of *hmgr* (HMGCoA reductase) gene, the key regulator of solasodine biosynthetic pathway (Shilpha et al., 2015). MJ at 25 μM promoted the expression of *PAL* genes by sevenfold on day 16 of elicitor treatment in *Ocimum tenuiflorum* suspension cell cultures (Vyas and Mukhopadhyay, 2018). The maximum yields of alkaloids and the highest levels of the expression of biosynthetic genes such as strictosidine synthase, geissoschizine synthase, deacetylvindoline acetyltransferase, and peroxidase were observed under 100 μM MJ in combination with 100 μM of AgNO_3_ after 7 days in *Catharanthus roseus in vitro* propagated shoots (Paeizi et al., 2018). Up-regulation of critical genes involved in the rubber biosynthesis pathway was exhibited by MJ treated *Hevea brasiliensis* barks (Liu et al., 2018). Soybean cell cultures treated with MJ treatment had the most significant effect on the expression of isoflavonoid biosynthesis genes (Jeong et al., 2018).

The simultaneous elicitation of MJ and putrescine in *Catharanthus roseus* shoots resulted in the up-regulation of signaling and biosynthetic genes of alkaloids production (Khataee et al., 2019). The relative expression levels of phenylpropanoid pathway genes, such as PAL, C4H, 4CL, and HPPR in the tyrosine-derived pathways were increased in MJ elicited hairy root cultures of *Mentha spicata* in comparison to untreated controls (Yousefian et al., 2020). The selected genes in the tanshinone and phenolic acid biosynthetic pathways were up-regulated with MJ elicitation in hairy root cultures of *Salvia przewalskii* (Li et al., 2020). Transcriptome analysis of MJ treated *Carthamus tinctorius* demonstrated the up-regulation of flavonoid biosynthesis pathway genes (Chen et al., 2020). MJ elicited callus cultures of *Capparis spinosa* showed the highest rutin content and increased expression patterns of rutin biosynthesis genes (Kianersi et al., 2021). Hairy root cultures of *Scutellaria bornmuelleri* exhibited enhanced expression of two important genes involved in the flavonoid biosynthesis pathway (Gharari et al., 2020). Besides, Table 2 presents a glimpse of the most recent reports correlated with gene expression and jasmonate elicitation.

**Table 2:** Reports on influence of jasmonates on gene expression during elicitation of secondary metabolites.

Plant species	Concentration of jasmonate used	Effect of elicitation on gene expression	Reference
*Punica granatum*	100 μM MJ	Enhanced expression of metabolic genes and transcription factors of tannin, flavonoid, and phyto-oxylipin pathways	Chang et al. (2021)
*Taraxacum kok-saghyz*	100 μM MJ	Increased expression of inulin biosynthetic pathway genes	Karimi et al. (2021)
Marine microalga *Isochrysis* sp.	150 μM MJ	Increased expression of the fatty acid desaturase gene Δ6Des	Ayothi et al. (2021)
Thyme sp.	100 μM MJ	Increased expression patterns of γ-terpinene synthase genes	Kianersi et al. (2021)
*Lycoris longituba*	75 μM MJ	Enhanced expression of Gal biosynthesis pathway genes and genes in the JA synthesis and JA signaling pathways	Li et al. (2021)
*Castilleja tenuiflora*	100 μM MJ	Upregulation of metabolite biosynthesis related genes such as *Cte-PAL1, Cte-CHS1*, *Cte-DXS1,* and *Cte-G10H*	Rubio-Rodríguez et al. (2021)
*Papaver bracteatum*	0.5 mM MJ	Increased expression of berberine bridge enzyme and norcoclaurine synthase genes	Esfahani et al. (2021)
*Andrographis paniculata*	25 μM MJ	Upregulation of biosynthetic genes of andrographolide	Srinath et al. (2021)
*Taxus baccata*	100 μM MJ	Increased levels of expression of DBTNBT gene, followed by ABC and BAPT genes	Kashani et al. (2022)
*Prunella vulgaris*	100 μM MJ	INCREASED expression of rosmarinic acid biosynthesis pTHWy genes such as *PvHPPR*, *PvPAL*, *PvC4H*, *Pv4CL1*, *Pv4CL2*, and *PvCYP98A101*	Ru et al. (2022)

Transcription factors are promising metabolic engineering targets due to their ability to regulate the transcription of multiple biosynthetic pathway genes. Jasmonate elicitation results in activation of TFs which regulate gene expression through specific binding to cis-acting elements in the promoters of target genes and so results in JAs-induced accumulation of secondary metabolites. These TFs belong to different families, including AP2/ERF, bHLH, MYB and WRKY TFs (De Geyter et al., 2012). De Geyter et al. (2012) and Zhou and Memelink (2016) provided in-depth reviews of various families of TFs and the effects of JAs on them. Some important studies published later are highlighted therein.

In *Vitis vinifera*, combined elicitation by MJ and cyclodextrins provoked the activation of additional regulatory pathways involving the upregulation of MYB15, NAC and WRKY transcription factors, protein kinases and calcium signal transducers, which in turn resulted in a greater trans-resveratrol production (Almagro et al., 2014). Induced by MJ treatment, the expression of a large number of genes involved in phenylpropanoid biosynthesis and many genes encoding transcription factors such as cytochrome P450s, glycosyltransferases, methyltransferases, and transporters in *Salvia sclarea* (Hao et al., 2015). MJ has been shown to activate the promoters of Ethylene Response Factor (ERF) family transcription factors such as ERF29, ERF210, and ERF199 at the nicotine-regulatory locus *NICOTINE2* in *Nicotiana tabacum*. These transcription factors have been shown to be most effective in controlling nicotine biosynthetic pathway genes (Shoji and Hashimoto, 2015). Many genes encoding transcription factors belonging to the ERF, bHLH, MYB, and WRKY families also responded and were upregulated due to MJ elicitation in *Gentiana macrophylla* (Cao et al., 2016). Sharma et al. (2019) reported the inducible nature of transcription factor *WsMYC2* in response to MJ elicitation in *Withania somnifera*. They have also witnessed the involvement of *WsMYC2* in the biosynthesis of triterpenoid withanolides and in inducing phytosterol *via* key pathway genes. MJ application in pear calli significantly enhanced flavonoid accumulation and upregulated the expression of the flavonoid biosynthesis pathway structural genes (*PcCHS, PcCHI, PcF3H, PcDFR, PcANS, PcANR2a*, and *PcLAR1*). In addition to *PcMYB10*, which is a known positive regulator of anthocyanin biosynthesis in pear, several novel MYB candidates that may regulate flavonol and proanthocyanidin biosynthesis were revealed (Premathilake et al., 2020).

### Elicitation of bioreactor-based cultures

Mass culturing of plant cells and tissues along with an enhanced accumulation of phytochemicals in bioreactors is the next step in large-scale, and commercial production of plant-based metabolites. In recent years, reports on bioreactor-based plant cultures have been blooming. Elicitation of bioreactor cultures further results in a large-scale production of plant secondary metabolites. Investigations on MJ elicitation in 5-L bioreactor suspension cultures of *Panax ginseng* demonstrated the highest ginsenoside accumulation at 200 μM (Thanh et al., 2005). A single 200 μM MJ treatment of *Panax ginseng* roots increased ginsenoside accumulation in airlift bioreactors (Ali et al., 2006). Mass cultivation of *Silybum marianum* hairy roots in a bioreactor resulted in the highest production of silymarin after MJ treatment (100 μM) for 3 weeks (Rahimi et al., 2012). In a pilot-scale bioreactor of 500 L capacity, elicitor-treated (100 μM MJ) adventitious roots of *Echinacea angustifolia* resulted in the maximum accumulation of total phenolics, total flavonoids, and total caffeic acid derivatives. Among the caffeic acid derivatives, the accumulation of echinacoside is approximately threefold more in MJ-treated adventitious roots than in non-MJ-treated roots cultured in bioreactors (Cui et al., 2013). MJ increased the scopolamine productivity by 146% in hairy root cultures of *Hyoscyamus niger* grown in a hybrid bubble column/spray bioreactor (Jaremicz et al., 2014). Surprisingly, adventitious root cultures of *Tripterygium wilfordii* grown in bubble column bioreactors showed 3.55-fold, 49.11-fold, and 10.40-fold increased accumulation of triptolide, wilforgine, and wilforine, respectively, upon MJ elicitation (Miao et al., 2014). *In vitro* shoots of *Vitis flexuosa* cultured in continuous immersion bioreactors recorded a greater quantity of total phenolics after MJ elicitation combined with manipulation of the NH_4_^+^ and NO_3_^–^ ratio (Park et al., 2015). The adventitious roots of *Eleutherococcus koreanum* in airlift bioreactors subjected to MJ treatment for 1 week showed the highest production of five target bioactive compounds (i.e.) eleutherosides B and E, chlorogenic acid, and total phenolics and flavonoids (Lee et al., 2015).

Treatment with 50 μM of MJ stimulated galanthamine and lycorine biosynthesis in *Leucojum aestivum* and *L. aestivum* bioreactor cultures (Ptak et al., 2017). Enhanced production of thapsigargin was achieved by growing *in vitro* shoots of *Thapsia garganica* in temporary immersion bioreactors using reduced nutrient supply in combination with MJ elicitation treatments (López et al., 2018). Cultivation of MJ elicited *Ocimum basilicum* suspension culture in bioreactors improved the cumulative productivities of betulinic acid, ursolic acid, and oleanolic acid (Pandey et al., 2019). The contents of total flavonoids and flavonoid monomers, including quercetin, kaempferide, epicatechin gallate, quercetin-3-O-glucose, and kaempferol-3-rutinoside, were significantly improved by MJ treatment in *Orostachys cartilaginous* cell cultures in balloon type bioreactors (Hao et al., 2020). *Pueraria candollei* var. *mirifica* cell suspension cultures in an airlift bioreactor exhibited enhanced production of deoxymiroestrol by MJ elicitation (Udomsin et al., 2020). *In vitro* seedlings of *Dendrobium nobile* grown in a temporary immersion bioreactor system were elicited with 10 μM MJ and resulted in a fivefold increased production of alkaloids (Zhang et al., 2022).

## Conclusion and future perspectives

Secondary metabolite biosynthesis impulse under the action of phytohormones in general and JAs, in particular, is crucial for plant growth, development, and defense in a stressed environment. So far, tremendous efforts have led to the elucidation of some of the key genes involved in the physiological mechanisms deployed by the plant to grow and survive. The JAs-elicited plant secondary metabolism machinery involved several transcription factors, including AP2/ERFs (Verpoorte et al., 2000), WRKYs (Wang et al., 2007), bHLHs (Zhang et al., 2011), and R2R3-MYBs (Shan et al., 2009). For example, the interaction network with synthesis enzymes has been under elucidation in the plant model *A. thaliana* and some medicinal plant systems including *Catharanthus roseus*, *Nicotina tabacum* and *Artemisia annua* (De Geyter et al., 2012).

A better understanding of the accumulation of plant metabolites is garnering more attention due to the increased interest in naturally produced secondary metabolites in human health, plant protection, and nutraceutical enriched foods. To extend the application field, we suggested the inclusion of medicinal orphan crops that also represent a secondary metabolite gold mine that deserves to be investigated. Among those orphan crops, the transcriptional comprehension of certain secondary metabolite synthesis has been initiated (Kang et al., 2020; Hu et al., 2021; Wang et al., 2021a,b) paving the way for not only effective JA triggered health-oriented metabolic engineering but also engineered crop protection against adverse biotic and abiotic stresses. Under the ongoing environmental change status, JAs’ mediated metabolite elicitation machinery needs further investigation in diverse plant systems. Moreover, the synthetic biology approach offers a novel path to improve the production ratio of secondary metabolites and deserves more attention from scientists.

## Author contributions

S-IS and SP conceived the review. S-IS, KR, ML, and SB wrote the manuscript. S-IS, YZ, JS, and MR made a critical revision of the review. SP, KR, ML, ST, and YZ performed the literature search. S-IS, ST, and SB prepared figures and tables. All authors contributed to the article and approved the submitted version.

## Funding

This study was carried out with the support of Research Program for Agricultural Science and Technology Development (project no. PJ01672604), Rural Development Administration and 2022 Post-Doctoral Fellowship Program (project no. PJ01494301 and PJ01672604; SP and ST), and National Institute of Agricultural Sciences, Rural Development Administration, Korea.

## Conflict of interest

The authors declare that the research was conducted in the absence of any commercial or financial relationships that could be construed as a potential conflict of interest.

## Publisher’s note

All claims expressed in this article are solely those of the authors and do not necessarily represent those of their affiliated organizations, or those of the publisher, the editors and the reviewers. Any product that may be evaluated in this article, or claim that may be made by its manufacturer, is not guaranteed or endorsed by the publisher.
